# Bifurcated Aneurysm Location Predicts In-Stent Stenosis After Neuroform-EZ Stent-Assisted Coiling for Intracranial Aneurysm

**DOI:** 10.3389/fneur.2022.873014

**Published:** 2022-05-13

**Authors:** Wei You, Junqiang Feng, Huijian Ge, Hengwei Jin, Peng Liu, Youxiang Li, Yuhua Jiang, Xinke Liu

**Affiliations:** ^1^Department of Interventional Neuroradiology, Beijing Neurosurgical Institute and Beijing Tiantan Hospital, Capital Medical University, Beijing, China; ^2^Beijing Engineering Research Center, Beijing, China

**Keywords:** neuroform-EZ stent, stent-assisted coiling, in-stent stenosis, intracranial aneurysm, bifurcated aneurysm

## Abstract

**Background and Purpose:**

The Neuroform EZ stent system (Boston Scientific Corporation, Fremont, CA, United States) is a fourth-generation intracranial aneurysm stent designed specifically for the cerebrovasculature to support aneurysm treatment. In this study, we analyzed our consecutive series of patients with aneurysm treated with the Neuroform EZ stent, with special attention to the occurrence of in-stent stenosis (ISS).

**Methods:**

A retrospective review of our center's electronic database was conducted to identify all patients with intracranial aneurysms who underwent aneurysm treatment with the Neuroform EZ stent between January 2016 and October 2018. Patients with at least one digital subtraction angiography (DSA) follow-up in our hospital were enrolled in this study. In-stent stenosis (ISS) was graded as mild (<2–5%), moderate (25–50%), or severe (>50%).

**Results:**

The study included 114 patients (78 women, 68.4%; median age 57.2 ± 9 years) with a total of 116 aneurysms. Of the 116 lesions, 20 were identified with ISS (17.2%) at a mean follow-up of 6.9 ± 1.7 months, and ISS was mild in 30% (6/20), moderate in 50% (10/20), and severe in 20% (4/20). No patients were symptomatic or required further intervention. Patients who developed ISS were younger than those without ISS (52.6 ± 7.8 vs. 57.9 ± 9; *p* = 0.016). The proportion of aneurysms located at the artery bifurcation was significantly higher in patients with stenosis than located at the sidewall artery (37.9 vs. 10.3%; *p* = 0.002). In the multivariable analysis, the patients' age (OR = 0.94; 95% CI 0.88–0.998; *p* = 0.02) and bifurcated aneurysm location (OR = 4.59; 95% CI 1.54–13.67; *p* = 0.006) were independent predictors of ISS.

**Conclusions:**

In this retrospective study, the incidence of ISS after Neuroform EZ stent placement was 17.2%, and all the ISS cases were asymptomatic. Patients with younger age and bifurcated aneurysm location are more likely to develop ISS. Although Neuroform EZ stent is particularly suitable for bifurcated aneurysms, the ISS for this location should be focused upon.

## Introduction

The Neuroform Microdelivery Stent System (Stryker Neurovascular, Fremont, CA, United States) was the first device approved by the Food and Drug Administration (FDA) in 2002 to treat intracranial aneurysms. Subsequent product iterations, especially the Neuroform EZ stent system (NF-EZ), were approved in 2010. The NF-EZ stent is an excellent choice for most intracranial aneurysms, specifically those with tight bends in the parent vessel or aneurysms located at the artery bifurcation (1).

Although much attention has been paid to the cure rate, ischemic complications, and hemorrhage of aneurysms, delayed complications of in-stent stenosis (ISS) have not been extensively studied. The reported incidence of ISS after stent-assisted coiling (SAC) depends on the type of stent, and ranges from 2.3 to 17.9% (2–7). However, there is lack of data on ISS after NF-EZ stenting for an aneurysm. In this study, we analyzed our consecutive series of patients with aneurysm treated with NF-EZ SAC, with special attention to the occurrence of ISS.

## Methods

### Study Population

We retrospectively reviewed patients with intracranial aneurysms who received SAC therapy with the NF-EZ stent at the neurological intervention center of our hospital between January 2016 and October 2018. Patients were included in this study if they underwent at least one cerebral digital subtraction angiography (DSA) follow-up examination in our hospital. Patient demographics, aneurysm characteristics, and clinical and angiographic outcomes were reviewed. This study was approved by the institutional review board of our hospital. The consent of each patient was obtained before study enrollment.

### Endovascular Procedure

Patients who were considered for treatment were receiving antiplatelet pharmacotherapy (100 mg aspirin and 75 mg clopidogrel daily) for at least 5 days before the procedure, including on the day of the procedure. Patients with preoperative subarachnoid hemorrhage were provided with an antiplatelet medication following the procedure. All the procedures were performed under general anesthesia *via* a femoral approach. When the stent was released completely, visualization of the 4 platinum marker bands at both ends of the stent affirmed the proximal and distal positions of the stent in the parent vessel. Coil embolization was mainly performed with the introduction of a microcatheter through the cell of the stent. Following the procedure, dual antiplatelet therapy was maintained for 6 months, and aspirin was continued indefinitely thereafter.

### Assessment of ISS

ISS is defined as any loss in the diameter of the parent artery around the stent implantation segment in angiographic follow-up images. The degree of stenosis was evaluated based on the European Carotid Surgery Trial standard (8). The percentage of stenosis was calculated as 1–(narrowest vessel diameter within the stenosis/maximum vessel diameter of the artery) ×100%. ISS was graded as mild (<25%), moderate (25–50%), or severe (>50%). Additionally, ISS was classified as proximal, middle, or distal based on location, and as focal or diffuse based on whether the stenosis extended more than 10 mm. The assessment of ISS was performed by a neuroradiologist with 3 years of experience through DSA follow-up images, and the assessments were reviewed by a senior neuroradiologist.

### Statistical Analysis

Data are presented as frequency for categorical variables and as the mean with range for continuous variables. Chi-square test or Fisher exact test was conducted to analyze categorical variables, and independent samples *t*-test was conducted to analyze continuous variables. Binary logistic regression analysis was conducted to identify significant independent predictors of severe ISS. Variables that were found to be significant at the level of 0.1 in univariate analysis or based on clinical relevance were subjected to binary logistic regression analysis. The results are presented in the form of odds ratio (OR) and corresponding 95% confidence interval (CI). A value of *p* < 0.05 was considered to indicate statistical significance. A receiver operating characteristic (ROC) curve was used to analyze the performance of a logistic regression classification model. We performed statistical analysis and plotted the figures using SPSS and GraphPad software.

## Results

Of the 301 patients that received SAC treatment using NF-EZ stent in our center between January 2016 and October 2018, 114 (78 women, 68.4%; median age 57.2 ± 9 years) with a total of 116 aneurysms had at least one DSA follow-up examination and were eventually enrolled in this study. Demographics, aneurysm characteristics, and angiographic outcomes of the patients are presented in Table 1.

**Table 1:** Univariate and multivariate logistic analyses in association with in-stent stenosis (ISS).

**Variables**	**ISS (*n* = 20)**	**Non-ISS (*n* = 95)**	**Total (*n* = 115)**	**Univariate**	**Multivariate**	
				***p***	***p***	**OR (95%CI)**
**Baseline demographics and clinical characteristics**						
Female	12 (60%)	66 (68.8%)	78 (67.2%)	0.6		
Mean (range) age, years	52.6 ± 7.8	57.9 ± 9	57 ± 9	0.02^*^	0.04*^†^*	0.94 (0.88–0.998)
**Co-morbiditied**						
Hypertension	11 (55%)	60 (62.5%)	71 (61.2%)	0.62		
Diabetes	2 (10%)	11 (11.5%)	13 (11.2%)	1		
Hyperlipidemia	0 (0%)	5 (5.2%)	5 (4.3%)	0.59		
Current smoking	6 (30%)	12 (12.5%)	18 (15.5%)	0.08	0.28	2 (0.57–7.04)
Alcohol abuse	3 (15%)	14 (14.6%)	17 (14.7%)	1		
Symptomatic presentation of IA	13 (65%)	55 (57.3%)	68 (58.6%)	0.62		
Reptured (history of SAH)	3 (15%)	7 (7.3%)	10 (8.6%)	0.37		
Previous treatment of IA	0 (0%)	5 (5.2%)	5 (4.3%)	0.59		
**Aneurysm characteristics**						
Saccular aneurysm	19 (95%)	88 (91.7%)	107 (92.2%)	0.7		
Aneurysm size (max length, mm)	6.52 ± 3.49	6.39 ± 3.82	6.41 ± 3.75	0.89		
Aneurysm neck size (mm)	4.28 ± 2.13	4.61 ± 2.37	4.56 ± 2.32	0.57		
Bifurcation aneurysm	11 (55%)	18 (18.8%)	29 (25%)	0.002^*^	0.006*^†^*	4.59 (1.54–13.67)
Posterior circulating aneurysm	7 (35%)	16 (16.7%)	23 (19.8%)	0.07	0.43	1.6 (0.5–5.11)
**Angiographic outcome**						
RROC immediately				0.36		
1	10 (50%)	62 (64.6%)	72 (62.1%)			
2	5 (25%)	14 (14.6%)	19 (16.4%)			
3	5 (25%)	20 (20.8%)	25 (21.6%)			
RROC at first FU				0.42		
1	14 (70%)	76 (79.2%)	90 (77.6%)			
2	2 (10%)	10 (10.4%)	12 (10.3%)			
3	4 (20%)	10 (10.4%)	14 (12.4%)			

*IA, intracranial aneurysm; RROC, Raymond-Roy occlusion classification; FU, follow-up;*

* *significant difference in univariate analysis*;

† *significant difference in multivariate analysis*.

The mean max length of the aneurysms and mean aneurysm neck size were 6.4 ± 3.8 and 4.6 ± 2.3 mm, respectively. Ruptured aneurysms accounted for 8.6% (10/116) of the cases. Five aneurysms had been previously treated, four by coiling and one by clipping. Most of the aneurysms were located in the internal carotid artery (57.8%, 67/116), 6.9% (8/116) in vertebral arteries, 12.9% (15/116) in the basilar and other posterior cerebral arteries, and 22.4% (26/116) in the distal circle of Willis (including middle cerebral artery, anterior cerebral artery, anterior communicating artery, and posterior communicating artery). Twenty-five (29/116) percent of the aneurysms were located at the artery bifurcation and 80.2% (93/116) in the anterior circulation. Bifurcation aneurysms were located at the middle cerebral artery bifurcation (44.8%, 13/29), basilar artery tip (37.9%, 11/29), and anterior communicating artery (17.2%, 5/29).

All the patients received SAC therapy for aneurysm. Multiple stenting was performed using the Y-configuration for three cases (2.6%), in which the aneurysms were all located at the tip of the basilar artery. Immediate complete and nearly complete aneurysm occlusions were achieved in 78.4% of the cases (grade 1, 62.1%; grade 2, 16.4%). Follow-up angiography (mean follow-up duration 7.5 ± 2.4 months) showed complete and nearly complete aneurysm occlusions in 87.9% of the cases (grade 1, 77.6%; grade 2, 10.3%). Retreatment by coiling was performed in two cases of recurrent aneurysm. Treatment-related complications were observed in 5/116 cases (4.3%) in the periprocedural period. There were two cases of aneurysm rupture during the treatment procedure, one case of parenchymal hemorrhage and one case of SAH; the rest of the infarctions occurred after the treatment procedure (<24 h). Transient deficits were observed in 4/5 cases (mRS <2) and permanent deficits in one case (mRS = 4).

**Figure 1:**
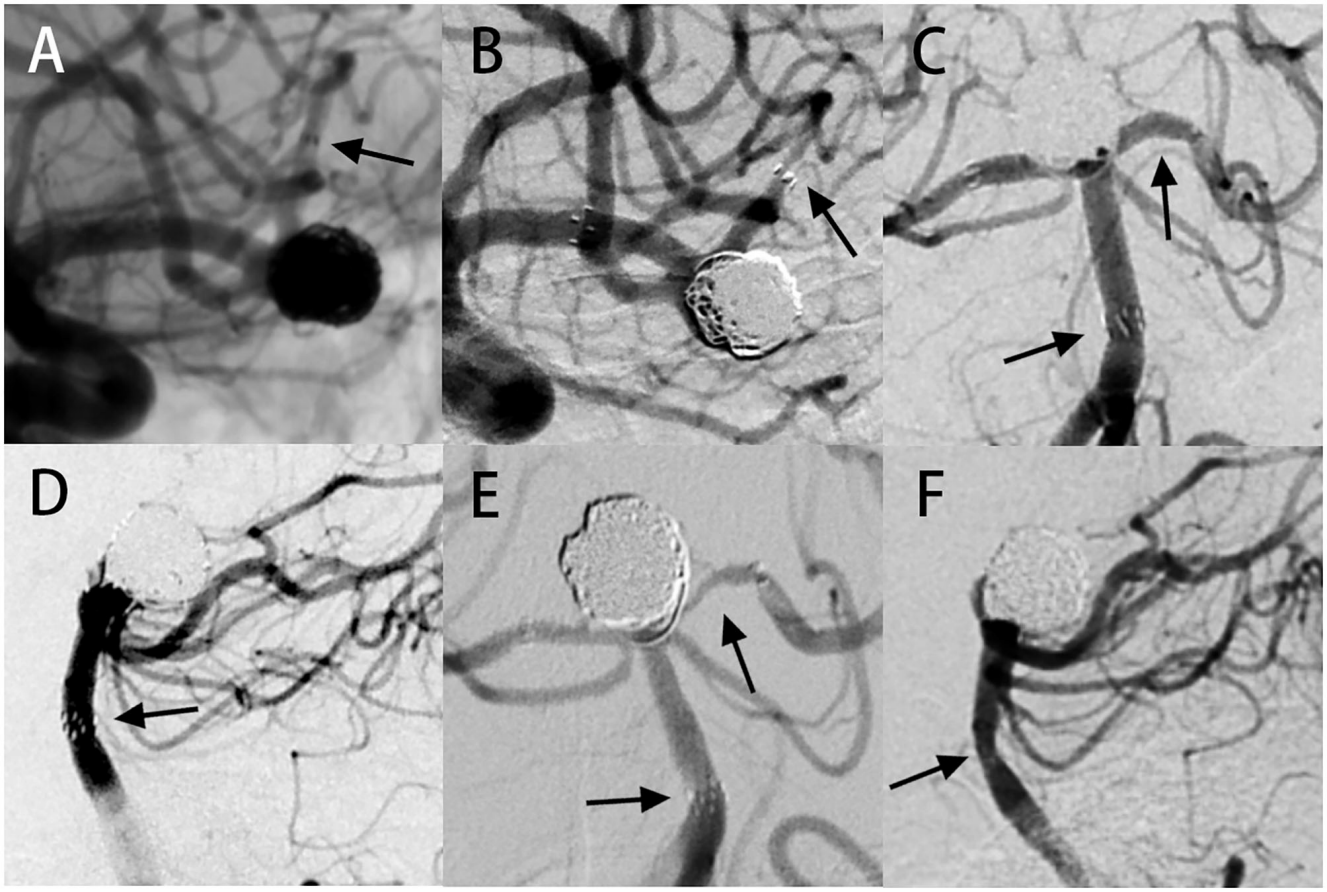
In-stent stenosis case presentation. Case 1: **(A)** digital subtraction angiography (DSA) images showing an aneurysm located at the bifurcation of the left middle cerebral artery. Stent-assisted coiling (SAC) therapy was performed with the NF-EZ stent. **(B)** Angiography follow-up at 13 months showing severe ISS at the distal end of the stent. Case 2: **(C,D)** DSA images showing an aneurysm at the tip of the basilar artery. Two NF-EZ stents were used for the “Y” type SAC operation. **(E,F)** Six-month follow-up angiography showing multiple ISS, including **(E)** severe ISS in the left posterior cerebral artery at the distal of the stent and **(F)** moderate ISS in the basilar artery at the proximal end of the stent. The arrow shows the location of ISS.

**Figure 2:**
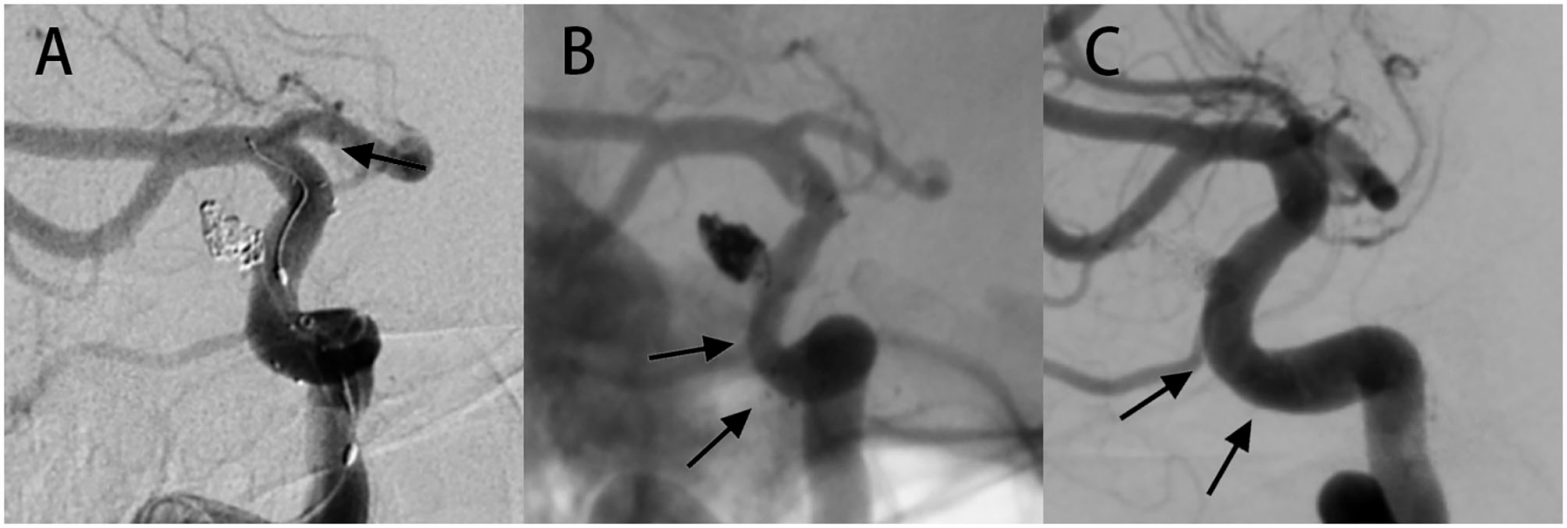
Case of ISS showing complete resolution during long-term angiographic follow-up. **(A)** A 52-year-old female had an aneurysm in the right internal carotid artery (ICA) a paraophthalmic aneurysm, and was treated with SAC therapy using the NF-EZ stent. On the immediate postoperative image, the marker at both ends of the stent showed that the stent was in the proper position. **(B)** Follow-up image at 5 months revealing extensive mild ISS. The patient was asymptomatic and continued to undergo dual antiplatelet therapy. **(C)** Angiography at 29 months follow-up revealing complete resolution of ISS. The arrow shows the location of ISS.

There were 17.2% (20/116) cases identified with ISS at the first angiographic follow-up (mean follow-up duration 6.9 ± 1.7 months). The radiological and clinical characteristics of 20 ISS cases are shown in Table 2 and examples of ISS are show in Figure 1. ISS was mild in 30% of the cases (6/20), moderate in 50% of the cases (10/20), and severe in 20% of the cases (4/20). Stenosis was diffuse in 55% of the cases (11/20) and focal in 45% of the cases (9/20). Stenosis was located at the proximal end of the stent in 45% of the cases (11/20), in the middle in 15% of the cases (3/20), and at the distal end in 30% of the cases (6/20). Sidewall and bifurcation aneurysms accounted for 45 and 55% of the ISS cases, respectively. Three ISS cases (15%) had a mean angiography follow-up time of 18.3 ± 5.5 months, and showed complete resolution in one of the cases (Figure 2) and stable condition in the other two. None of the patients with ISS were symptomatic or required further intervention.

**Table 2:** Radiological and clinical characteristics of the ISS cases.

**No**	**Age (y)**	**Sex**	**Stensois percentage**	**Degree of ISS**	**Aneurysm location**	**Time to stenosis (mo)**	**ISS type**	**Final FU (mo)**	**Prognosis**
1	49	M	14	Mild	MCA bif	6	Diffuse, Middle	N/A	
2	47	F	18	Mild	ICA	7	Diffuse, Distal	N/A	
3	43	M	18	Mild	BA tip	8	Focal, Proximal	N/A	
4	50	M	19	Mild	AcomA	7	Focal, Proximal	N/A	
5	43	F	23	Mild	ICA	6	Focal, Distal	13	Stable
6	59	F	24	Mild	BA tip	6	Diffuse, Distal	N/A	
7	41	F	25	Moderate	BA trunk	7	Diffuse, Distal	18	Stable
8	61	F	26	Moderate	MCA bif	7	Diffuse, Proximal	N/A	
9	55	M	28	Moderate	ACA	6	Focal, Proximal	N/A	
10	61	F	29	Moderate	MCA bif	8	Diffuse, Distal	N/A	
11	52	F	30	Moderate	ICA	5	Diffuse, Middle	29	CR
12	45	M	35	Moderate	PCA	5	Focal, Proximal	N/A	
13	41	F	35	Moderate	ICA	7	Diffuse, Proximal	N/A	
14	57	F	40	Moderate	BA tip	7	Focal, Proximal	N/A	
15	61	M	42	Moderate	MCA bif	7	Diffuse, Proximal	N/A	
16	60	F	44	Moderate	ICA	6	Focal, Middle	N/A	
17	50	F	65	Severe	BA tip	6	Focal, Proximal	N/A	
18	40	M	67	Severe	ACA	8	Diffuse, Proximal	N/A	
19	62	F	73	Severe	MCA bif	13	Focal, Proximal	N/A	
20	57	M	1	Severe	BA tip	6	Diffuse, Distal	N/A	

*N/A, not applicable; F, female; M, male; y, years; bif, bifurcation; mo, month; FU, follow-up; CR, completely resolved; MCA, middle cerebral artery; ICA, internal carotid artery; BA, basilar artery; ACA, anterior cerebral artery; PCA, posterior cerebral artery; AComA, anterior communicating artery*.

**Figure 3:**
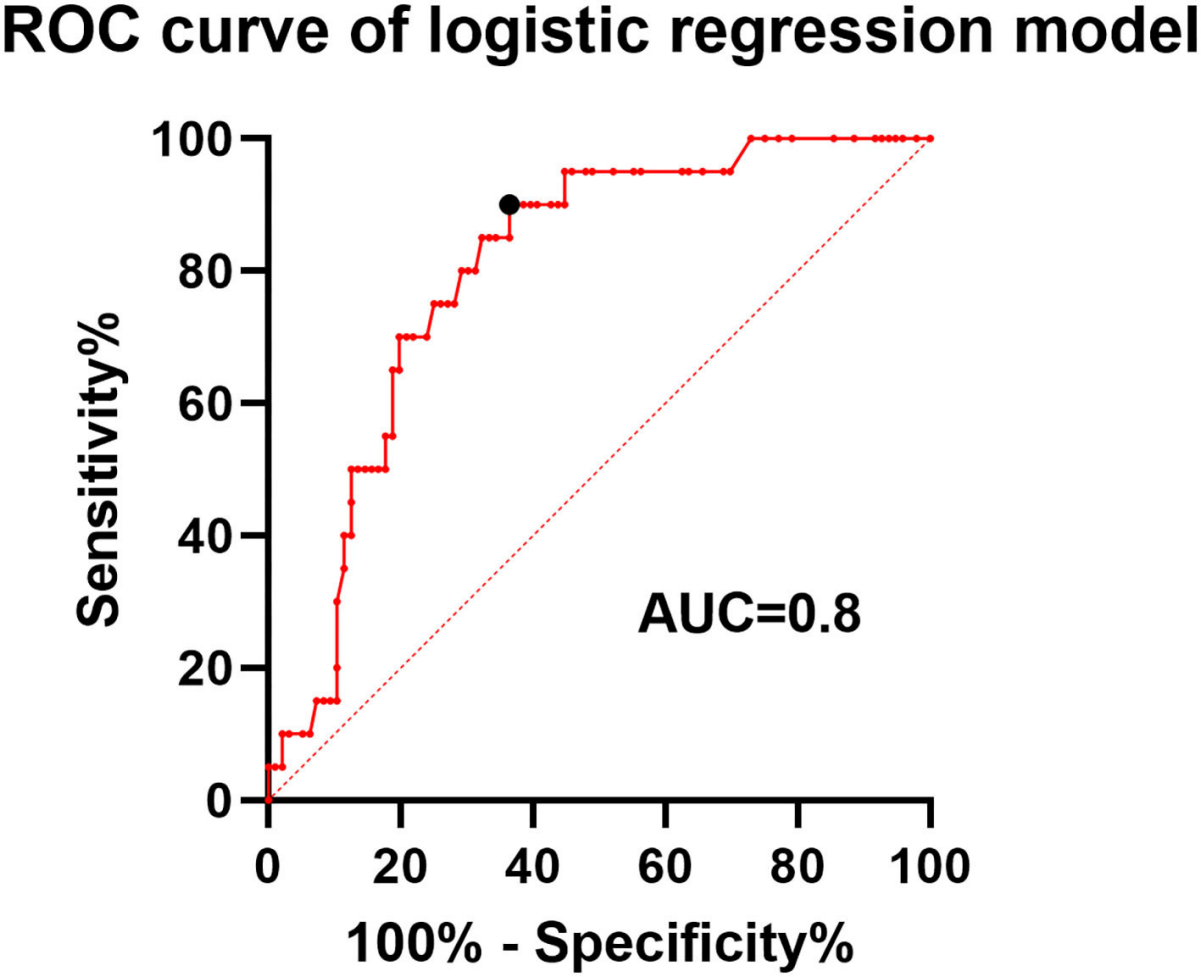
Receiver operating characteristic (ROC) curve of the logistic regression classification model. The area under the curve (AUC) was 0.8 with a sensitivity and specificity of 0.9 and 0.64 at the point of the cutoff value.

The univariate and multivariate logistic analyses, in association with ISS, are presented in Table 1. Patients who developed ISS were younger than those without ISS (52.6 ± 7.8 vs. 57.9 ± 9; *p* = 0.016). The proportion of aneurysms located at the artery bifurcation was significantly higher in patients with stenosis than those without stenosis (37.9 vs. 10.3%; *p* = 0.002). The proportion of patients with current smoking status (33.3 vs. 14.3%; *p* = 0.08) and posterior circulation aneurysm (30.4 vs. 14%; *p* = 0.072) was higher but not significantly in the ISS group than in the non-ISS group. In the univariate analysis, significant variables at the threshold of 10% were subjected to binary logistic regression analysis. In the multivariate analysis, the patients' age (OR = 0.94; 95% CI 0.88–0.998; *p* = 0.02) and bifurcated aneurysm location (OR = 4.59; 95% CI 1.54–13.67; *p* = 0.006) exhibited strong independent associations with ISS. Specifically, the younger patients and those with aneurysms at the artery bifurcation were more likely to develop ISS. ROC curve was used to analyze the performance of the logistic regression classification model, and the area under the curve (AUC) was 0.8 (Figure 3).

## Discussion

The occurrence of ISS after NF-EZ stenting for the treatment of aneurysms is not well-studied. We found that 17.2% of the patients in our study had a radiographically identifiable ISS, but no patient showed symptoms or required further intervention. The multivariate analysis identified younger age and bifurcated aneurysm location as independent predictors of ISS.

The reported incidence of ISS and symptomatic stenosis after SAC is 2.3–17.9 and 0–1.3%, respectively (2–7, 9). ISS incidence after Neuroform stent implantation is relatively low, mostly between 2.4 and 7.5% (2, 3, 10). In our study, the incidence of ISS after NF-EZ stenting was 17.2% (20/116), which was relatively higher than that previously reported. The difference in ISS incidence may be because of the difference in ISS criterion. Some suggest <25% narrowing as intimal hyperplasia (11, 12), while some suggest that only >50% narrowing is significant (3–5, 10, 13). Our high incidence rate (17.9%) would be reasonable considering that our criterion of ISS is below 25%. However, stenosis <25% should not be overlooked, as it is known that ISS is a dynamic phenomenon, and the observed progression of stenosis may indicate the need for timely intervention. In the report of Kim et al. (9), a patient with stenosis of <25% progressed to occlusion during long-term follow-up.

ISS is a well-known issue of endovascular stents, especially in the treatment of coronary artery and atheromatous diseases. Abnormal vascular remodeling and neointimal hyperplasia are considered to be the underlying causes of ISS (14). Disruption of endothelial function by vascular endothelial injury leads to proliferation of local smooth muscle cells, formation of neointimal tissue, and eventually ISS (15). Overlying the stent with the endothelium may lead to prolonged endothelialization, because the middle of the stent will be reached from the edge of the stent. Persistent neointimal hyperplasia in the stented segment may significantly compromise the parent vessel (3, 7, 16, 17). The longer time required for reendothelialization further stimulates more proliferation of smooth muscle cells at the edge of the stent, since the process continues as long as the endothelial layer is incomplete (18, 19).

The degree of neointimal hyperplasia and ISS after SAC may be related to the severity of endothelial injury during stenting and further manipulations that may affect the stability of the stent during the initial process (10). There is a striking difference in the incidence and natural history of ISS between balloon-expanded stents used for atheromatous disease and NF-EZ stent (20). The deployment of a balloon-expanded stent or balloon angioplasty for atheromatous disease will inevitably lead to disruption and denudation of the endothelium over the treated vascular segment and result in a relatively high rate of postoperative restenosis (32%) (20). Deployment of the NF-EZ stent causes much less injury than either angioplasty alone or deployment of a conventional balloon-mounted stent, because the self-expanding NF-EZ stent has a lower radial force (16) and does not require balloon angioplasty.

The Neuroform EZ stent system is a fourth-generation intracranial aneurysm stent with an integrated navigation guidewire, and is designed specifically for the cerebrovasculature to support aneurysm treatment. It has an open-cell design to allow for high navigability. The Neuroform EZ stent is an excellent choice for most intracranial aneurysms, especially those with tight bends in the parent vessel or those located at the artery bifurcation (1). The operator may obtain access with their choice of microwire and microcatheter and avoid the difficulty of *en bloc* advancement through curved anatomical structures. However, our results showed that patients with bifurcated aneurysms were more likely to develop ISS. Our finding that bifurcated aneurysm location is a predictor of ISS is not based on previous literature and may be due to complex procedures for bifurcation aneurysms. Compared with sidewall aneurysms, the treatment of bifurcated aneurysms requires delicate operation and high-skilled technology to protect the branch vessels, which inevitably increases operation time (21) and the risk of potential endothelial injury.

Younger age was also identified as an independent predictor of ISS. This finding is consistent with the study by Chalouhi et al. (22) on ISS after aneurysm stenting and the study by Turk et al. (23) on in-stent restenosis after stenting for atherosclerotic disease. They found that the rate of postoperative stenosis or restenosis was significantly higher in younger patients than in elderly patients possibly because the response of neointimal hyperplasia induced by stenting is more aggressive in younger patients. This hypothesis is supported by the study by Du et al. (24) who observed a significant reduction in neointimal growth after coronary stenting in elderly patients than in younger patients.

The prognosis of ISS is a benign process after stent implantation for aneurysm, since most ISS cases eventually become stable or improve (3, 9, 10, 25, 26). In this study, three ISS cases had long-term angiographic follow-up data, in which the stenosis was completely relieved in one of cases and stable in the other two. All the patients with ISS in this series remained asymptomatic during the follow-up period. Nevertheless, the occurrence of symptomatic stenosis in previously asymptomatic patients with ISS represents a considerable issue. Therefore, close observation and careful imaging follow-up are recommended for patients with ISS.

In most studies, ISS was identified during radiological follow-up by both magnetic resolution angiography (MRA) and catheter-selected angiography (3, 10, 27, 28). Although Prabhakaran et al. have suggested that MRA could be a promising screening method for detecting ISS, we chose DSA as the only follow-up imaging method in our research, since it is still the gold standard for cerebral disease. In addition, we only performed angiography for image evaluation, so image differences affected our assessment results less.

There are some limitations to this study. This is a single-center retrospective study that may increase the risk of selection bias. Some of the patients had an angiographic follow-up in local hospitals, leading to some loss in follow-up. Further exploration of large-scale cohorts with long-term follow-up data is needed.

## Conclusion

In this retrospective study, the incidence of ISS and its predictors were determined. We found that the incidence of ISS after NF-EZ stent placement was 17.9%, and that all the ISS cases were asymptomatic. Patients with younger age and bifurcated aneurysm location are more prone to developing ISS. Although the NF-EZ stent is particularly suitable for bifurcated aneurysms, our findings suggest that more attention should be paid to ISS after treatment.

## Data Availability Statement

The raw data supporting the conclusions of this article will be made available by the authors, without undue reservation.

## Ethics Statement

The studies involving human participants were reviewed and approved by Institutional Review Board at Tiantan Hospital. The patients/participants provided their written informed consent to participate in this study.

## Author Contributions

YL, YJ, and XL designed the study. WY and PL contributed to data collection. WY and JF performed the data analysis and drafted the manuscript. HJ and HG performed the revision of the current literature. All authors contributed to the manuscript and approved the final version.

## Funding

This study has received funding by the National Natural Science Foundation of China (82171289 and 81901197), the National Key Research and Development Program of China (2017YFB1304400).

## Conflict of Interest

The authors declare that the research was conducted in the absence of any commercial or financial relationships that could be construed as a potential conflict of interest.

## Publisher's Note

All claims expressed in this article are solely those of the authors and do not necessarily represent those of their affiliated organizations, or those of the publisher, the editors and the reviewers. Any product that may be evaluated in this article, or claim that may be made by its manufacturer, is not guaranteed or endorsed by the publisher.
